# Method comparison for cardiac image registration of coronary computed tomography angiography and 3-D echocardiography

**DOI:** 10.1117/1.JMI.5.1.014001

**Published:** 2018-01-04

**Authors:** Tim Nordenfur, Aleksandar Babic, Ivana Bulatovic, Anders Giesecke, Elif Günyeli, Jonaz Ripsweden, Eigil Samset, Reidar Winter, Matilda Larsson

**Affiliations:** a KTH Royal Institute of Technology, Department of Medical Engineering, Stockholm, Sweden; b Karolinska Institute, Department of Clinical Sciences, Stockholm, Sweden; c GE Vingmed Ultrasound, Oslo, Norway; d University of Oslo, Department of Informatics, Oslo, Norway; e Karolinska Institute, Department of Molecular Medicine and Surgery, Stockholm, Sweden; f Karolinska Hospital, Department of Emergency Medicine, Stockholm, Sweden; g Danderyd Hospital, Department of Cardiology, Stockholm, Sweden; h Karolinska Institute, Department of Clinical Science, Intervention and Technology, Stockholm, Sweden

**Keywords:** image registration, coronary computed tomography angiography, three-dimensional echocardiography, coronary artery disease, myocardial strain

## Abstract

Treatment decision for coronary artery disease (CAD) is based on both morphological and functional information. Image fusion of coronary computed tomography angiography (CCTA) and three-dimensional echocardiography (3DE) could combine morphology and function into a single image to facilitate diagnosis. Three semiautomatic feature-based methods for CCTA/3DE registration were implemented and applied on CAD patients. Methods were verified and compared using landmarks manually identified by a cardiologist. All methods were found feasible for CCTA/3DE fusion.

## 1 Introduction

Coronary artery disease (CAD) remains the leading cause of death worldwide.^1^^,^^2^ It is caused by plaque deposition in one or multiple coronary arteries producing a narrowing of the lumen called stenosis. Obstructive stenoses restrict the coronary blood supply, which can inhibit normal myocardial function and cause ischemia. Possible treatment options for patients with suspected stable CAD include lifestyle change, medical therapy, percutaneous coronary intervention (PCI), and coronary artery bypass grafting (CABG).^3^ PCI and CABG are invasive revascularization procedures and expose patients to multiple risks, including vascular damage, contrast-induced renal failure, myocardial infarction, and sepsis.^4^

Many morphologically apparent stenoses are not functionally significant. Due to the risks associated with invasive treatment procedures, treatment of functionally insignificant stenoses should be avoided. In the FAME study, disregarding measurements of fractional flow reserve during PCI treatment increased the risk of death or myocardial infarction within 2 years by 50%.^5^ Therefore, the choice of treatment strategy is frequently based on both morphological and functional information.^3^^,^^5^^,^^6^^–^^8^

Morphological information can be noninvasively obtained from coronary computed tomography angiography (CCTA) as high-resolution three-dimensional (3-D) images of the coronary artery tree and stenoses.^7^ However, due in part to low temporal resolution, CCTA is a poor predictor of the functional significance of stenoses.^7^^,^^9^ Functional information can be noninvasively obtained from stress three-dimensional echocardiography (3DE) in which stress, induced by exercise or pharmacological agents, increases the flow demand in stenotic coronary arteries. The increased demand can induce ischemia, resulting in wall motion abnormalities that can be directly observed.^8^ Other noninvasive functional imaging modalities include single-photon emission computed tomography (SPECT),^10^ positron emission tomography (PET),^11^ and cardiac magnetic resonance imaging.^12^ Advantages of 3DE include low cost and lack of ionising radiation. The current gold standard for functional imaging in CAD is invasive coronary angiography,^8^ which carries greater risks of death and myocardial infarction than noninvasive alternatives.^13^ Furthermore, only one-third of coronary angiography investigations are followed up by revascularization procedures,^13^ suggesting that improved noninvasive imaging modalities could reduce patient exposure to catheterization and associated complications.

To allocate stenotic segments to functional defects, morphological and functional information is traditionally viewed side-by-side and integrated mentally, a time-consuming and error-prone process. Side-by-side interpretation is further complicated by large individual variation of the coronary artery tree anatomy and myocardial distribution territories.^14^^,^^15^ These difficulties can be alleviated by fusing morphological and functional information into a single image with a single spatial frame of reference. In a study comparing fused and side-by-side interpretations of CCTA and SPECT myocardial perfusion images, image fusion enabled confident assessment of the functional significance of most stenoses that could not be classified in images viewed side-by-side.^16^ Therefore, cardiac image fusion might reduce unnecessary exposure to invasive diagnostic procedures, ultimately reducing mortality in CAD patients.

Image fusion of CCTA and SPECT myocardial perfusion images enables more confident assessment of the functional significance of most stenoses than viewing images side-by-side.^16^ Therefore, cardiac image fusion might facilitate interpretation and reduce unnecessary exposure to invasive diagnostic procedures, ultimately reducing mortality in CAD patients.

Fusion of CCTA and 3DE has recently been shown to be feasible.^17^^,^^18^ In this paper, we describe three methods for automatic CCTA/3DE registration based on segmentation and alignment. These methods are validated and evaluated in patients with suspected CAD by comparison with manually identified landmarks. Note that this paper represents an extension of preliminary results published in conference proceedings.^19^

## 2 Methods

### 2.1 Patient Population

We enrolled 16 consecutive patients who were scheduled to undergo CCTA at Karolinska University Hospital (Stockholm, Sweden) by their treating physician to rule out CAD. All patients consented to undergoing 3DE. The study was approved by the local ethics committee (Etikprövningsnämnden, diary no. 2014/1437-31/3). Five patients were excluded due to poor image quality. The median age of included patients was 61 years, ranging from 17 to 74 years. One patient was female and 10 were male.

### 2.2 Data Acquisition

One 3-D volume from CCTA and one four-dimensional (4-D) volume (3-D + time) from 3DE were acquired for each patient. Ten patients underwent 3DE within 1 hour of CCTA. The remaining patient underwent 3DE 48 days later.

All CCTA examinations were performed on a 64-channel detector CT scanner (LightSpeed VCT XT; GE Healthcare, Milwaukee, Wisconsin) with a prospectively ECG-triggered scan protocol. The tube potential was 120 kVp, tube current 450 to 650 mA depending on body mass index, rotation time 350 ms, and detector collimation 64×0.625  mm. Nine scans were performed at 75% of the RR interval with a padding of ±100  ms. The remaining two patients were scanned at 35% of the RR interval due to heart transplantations. All scans were performed with the patient in supine position. The contrast media used for CCTA examinations was iodixanol (Visipaque 320  mg/ml; GE Healthcare, Little Chalfont, United Kingdom), administrated with a triple-phase individually dosed contrast media protocol, using a dual-head injector (Medrad, Stellant Dual Head Injector, Pittsburgh, Pennsylvania). The contrast media was injected based on bodyweight (500 mg I/kg) with a fixed injection time at 15 s, which resulted in an injection rate of 4 to 8  ml/s. This was followed by a 50-ml mixture of 40% contrast media and 60% saline and finally by a 50-ml saline chaser. In the absence of contraindications, patients received sublingual nitroglycerin (0.4 mg) 4 to 5 min before the scan to reduce the heart rate. Depending on the initial heart rate, they also received metoprolol (25 to 100 mg) per 1 h before scanning. Average patient heart rate during CCTA acquisition ranged from 33 to 66 beats per minute.

All 3DE examinations were performed by an experienced sonographer using a Vivid E9 ultrasound system (GE Vingmed Ultrasound, Horten, Norway) with a 4-D matrix-array transducer (4V-D) with 1.67-MHz center frequency. Datasets were stored digitally for offline analysis using EchoPac (version 112.0.2, GE Healthcare, Horten, Norway). 4-D (3-D + time) volumes were stitched from subvolumes obtained by ECG gating from four to six consecutive cardiac cycles during one breathhold. Acquisition parameter ranges were 60 deg to 65 deg sector angle, 11 to 16 cm depth of field, and 29 to 52 volumes per second. The probe was positioned for standard transthoracic apical four-chamber view with the patient in left lateral decubitus position. No medication was given. The average patient heart rates during 3DE acquisition ranged from 39 to 79 beats per minute.

### 2.3 Image Segmentation

Semiautomatic segmentation was performed in both CCTA and 3DE volumes using the real-time contour tracking library.^20^ A model of the left ventricle (LV) was manually initialized based on rough LV position and orientation. The model was automatically deformed based on edge detection of voxel intensities normal to the model surface. For each patient, segmentation resulted in the LV endocardial surface mesh as well as endocardial apex, mitral valve center, and LV outflow tract (LVOT) positions. CCTA volumes used for segmentation were exported from Advantage Workstation (Advantage Workstation, GE Healthcare) with longitudinal resolution of 0.6 mm and transverse resolution from 0.3 to 0.5 mm. 3DE volumes were exported from EchoPac with axial resolution from 0.5 to 0.7 mm and lateral and elevational resolution from 0.8 to 1.1 mm. Note that the landmarks found by the segmentation software were not the same as the manual initialization.

### 2.4 Image Registration

The 3DE volume was registered to the CCTA volume using three alternative methods: landmark distance minimization based on Procrustes alignment,^21^ endocardium iterative closest point (ICP) alignment,^22^ and three-chamber alignment. Each registration method estimated a rigid transformation based on segmented anatomical landmarks in the CCTA volume and the 3DE volume of matching cardiac phase, which was 75% of the RR interval in nine patients and 35% in two.

The segmented landmarks were mitral valve centers $M_{\mathrm{CCTA}}$ and $M_{3\mathrm{DE}}$, outflow tract centers $O_{\mathrm{CCTA}}$ and $O_{3\mathrm{DE}}$, and endocardial apices $A_{\mathrm{CCTA}}$ and $A_{3\mathrm{DE}}$. Furthermore, the endocardial surfaces were segmented and represented as point clouds $\left\{P_{\mathrm{CCTA},i}\right\}$ and $\left\{P_{3\mathrm{DE},i}\right\}$, i=1,…,642.

Landmark distance minimization: The mean squared distances between corresponding landmarks from the two volumes was minimized, i.e., rigid Procrustes analysis without scaling or reflection.^21^ Landmarks used were endocardial apex, mitral valve center, and LVOT. As such, the rigid transformation used was the matrix $T_{\mathrm{proc}}$ that minimized the sum

$${\left|A_{\mathrm{CCTA}}-T_{\mathrm{proc}}A_{3\mathrm{DE}}\right|}^{2}+{\left|M_{\mathrm{CCTA}}-T_{\mathrm{proc}}M_{3\mathrm{DE}}\right|}^{2}+{\left|O_{\mathrm{CCTA}}-T_{\mathrm{proc}}O_{3\mathrm{DE}}\right|}^{2}.$$

Endocardium ICP alignment: The segmented endocardial surfaces from CCTA and 3DE, 642 points each, were aligned using the ICP method: the two surfaces were initially registered using the landmark distance minimization described above. Each point on the 3DE surface was associated to the closest point on the CCTA surface. A new transform was obtained by minimizing the mean squared distance of these 642 point pairs. Points were reassociated and a new transform obtained until the process converged.

Three-chamber alignment: The three-chamber views were aligned by the unique rigid transformation such that

1. Mitral valve centers of both volumes coincided, i.e., $M_{\mathrm{CCTA}}=T_{3\mathrm{ch}}M_{3\mathrm{DE}}$.
2. The long axes, as defined from mitral valve center to endocardial apex, coincided, i.e., the points $A_{\mathrm{CCTA}}$, $M_{\mathrm{CCTA}}$, $T_{3\mathrm{ch}}A_{\mathrm{CCTA}}$, and $T_{3\mathrm{ch}}M_{\mathrm{CCTA}}$ were collinear.
3. The three-chamber planes, as defined by mitral valve centers, apices and LVOT, coincided, i.e., the points $A_{\mathrm{CCTA}}$, $M_{\mathrm{CCTA}}$, $O_{\mathrm{CCTA}}$, $T_{3\mathrm{ch}}A_{\mathrm{CCTA}}$, $T_{3\mathrm{ch}}M_{\mathrm{CCTA}}$, and $T_{3\mathrm{ch}}O_{\mathrm{CCTA}}$ were coplanar.

**Fig. 1:**
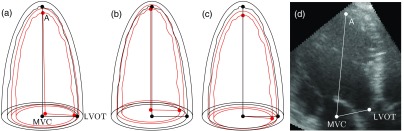
Schematic diagram of LVs from CCTA (black) and 3DE (red), registered using different registration methods: (a) landmark distance minimization, which minimizes the squared distances between apices (A), mitral valve centers (MVC), and left-ventricular outflow tracts (LVOT). (b) Endocardium ICP alignment, which minimizes distances between endocardial surfaces (inner wavy lines). (c) Three-chamber alignment, which aligns the mitral valve centers (MVC), long axes (A-MVC), and three-chamber planes (A-MVC-LVOT). (d) Demonstrates the landmarks overlaid on an echocardiographic three-chamber view.

Registration with the described methods required no manual intervention, other than the semiautomatic segmentation. All methods were implemented in C++ using the Visualization Toolkit.^23^ Figure 1 shows schematic diagrams of the three registration methods.

### 2.5 Anatomical Validation

Anatomical accuracy of the registration methods was quantitatively evaluated based on anatomical landmarks present in both volumes. An experienced cardiologist used a custom multiplanar reformatting visualization software to identify these LV anatomical landmarks. Note that the landmarks identified through this validation procedure are marked manually and are distinct from those detected in the semiautomatic segmentation step.

**Fig. 2:**
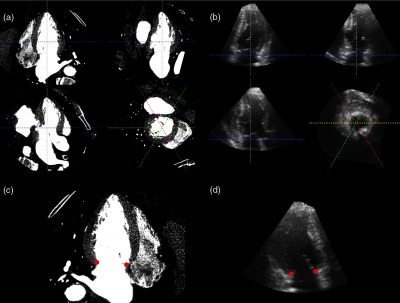
Screen captures from the custom software used to identify landmarks for evaluation of anatomical accuracy. (a) The interface used to identify standard views in a CCTA volume. (c) The three-chamber view rotated by 60 deg, which was one of the planes used to identify the mitral annulus. (b) and (d) The corresponding images from a 3DE volume.

The procedure performed for each volume was as follows:

The software presented three approximate long-axis and one short-axis view simultaneously. The user adjusted these views to obtain an optimally aligned three-chamber view. Figures 2(a) and 2(b) show the adjusted views from one 3DE and one CCTA volume. The software internally produced additional views by rotating the identified three-chamber view around the normal of the short-axis view. The user was presented with the three-chamber view rotated by 0 deg, 60 deg, and 120 deg consecutively. In each view, the user marked the endocardial apex. The user was presented with the three-chamber view and marked the center of the aortic valve. The user was presented with the three-chamber view rotated by 0 deg through 165 deg in 15-deg increments consecutively. In each view, the user marked the two points of the mitral annulus. Figures 2(c)–2(d) show the 60-deg view and identified points for one patient. Finally, the user was again presented with the three-chamber view rotated by 0 deg, 60 deg, and 120 deg consecutively. In each view, the user delineated the endocardium.

The aforementioned landmark identification workflow was performed first for each 3DE volume in a random order, then for each CCTA volume in a random order. For the 4-D volumes from 3DE, the cardiologist viewed the entire cine loop but marked the landmarks only in the time frame of cardiac phase matching the CCTA volume. The LV endocardial apex in a volume was taken as the mean of the three apical landmarks identified in different views and the mitral annular center position as the mean of the 24 mitral annulus landmarks.

**Fig. 3:**
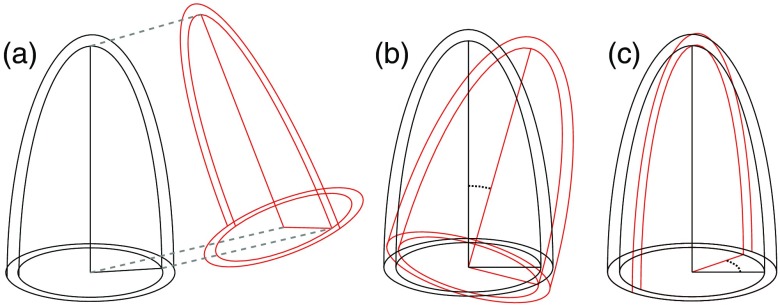
Schematic diagram of metrics used to quantify registration quality: (a) distances between mitral centers, aortic centers, and apices, (b) transverse-plane angle, and (c) long-axis angle.

Anatomical accuracy was quantified in each fused volume as the intermodality distances between corresponding anatomical landmarks [Fig. 3(a)]. Apex distance was defined as the distance from endocardial apex in the CCTA volume to endocardial apex in the 3DE volume. Mitral annular center distance and aortic valve center distance were defined correspondingly.

Furthermore, two intermodality angles were calculated to quantify LV rotation: long-axis angle and transverse-plane angle [Figs. 3(b)–3(c)]. The long axis in each modality was estimated as the line from endocardial apex to the mitral annular center. The long-axis angle was defined as the angle between the long axes from the two modalities. To estimate the transverse-plane angle, an average was first formed of the two long axes from 3DE and CCTA. The transverse-plane angle was defined as the angle between the aortic valve centers with respect to the average long axis.

Finally, two additional measures were used to assess endocardial surface similarity: the mean point-to-point distance was obtained by averaging the distances from each point on the segmented 3DE endocardial surface to the CCTA surface. Dice’s coefficient was obtained by considering the intersection between the three-chamber view, as identified by a cardiologist in the CCTA volume using the visualization software described above and the two segmented endocardial surfaces. The two intersections, the 3DE three-chamber LV area A and the CCTA three-chamber LV area B, were combined as follows to obtain Dice’s coefficient in the three-chamber view:

$$\mathrm{Dice}=2\frac{\left|A\cap B\right|}{\left|A|+|B\right|}.$$

The Wilcoxon signed-rank test was used to assess whether the quality measures vary between the registration methods presented. Tests were performed for each quality measure between each pair of registration methods for a total of 7×3=21 tests.

### 2.6 Interobserver and Intraobserver Variability

Variations in the manual initialization of segmentation could affect the final registration. To evaluate this effect, segmentation was repeated on the data of four patients. For each of these patients, both the CCTA and 3DE volumes segmentation was repeated once by the original operator and once by another operator. Segmentation was performed as described in Sec. 2.3. Analogously to the landmarks named in Sec. 2.4, this yielded additional points $\left\{M_{\mathrm{CCTA}}^{\prime },A_{\mathrm{CCTA}}^{\prime },O_{\mathrm{CCTA}}^{\prime }\right\}$ as segmented again by the original operator and $\left\{M_{\mathrm{CCTA}}^{\prime \prime },A_{\mathrm{CCTA}}^{\prime \prime },O_{\mathrm{CCTA}}^{\prime \prime }\right\}$ as segmented by another operator, as well as corresponding six points from the 3DE volumes.

Using the three-chamber alignment method, as described in Sec. 2.4, a rigid transform $T^{\prime }$ was estimated to register the points $\left\{M_{3\mathrm{DE}}^{\prime },A_{3\mathrm{DE}}^{\prime },O_{3\mathrm{DE}}^{\prime }\right\}$, i.e., the repeated segmentation of the 3DE volume by the original operator, with the points $\left\{M_{\mathrm{CCTA}},A_{\mathrm{CCTA}},O_{\mathrm{CCTA}}\right\}$, i.e., the original segmentation of the CCTA volume. The intraobserver variability in registration was quantified through comparison between the transforms obtained from original segmentation, T, and from repeated segmentation, $T^{\prime }$. Three landmark distance measures and two angle measures were used, analogously to the validation measures described in Sec. 2.5.

The landmark distances used were apex distance $\left|TA_{3\mathrm{DE}}-T^{\prime }A_{3\mathrm{DE}}^{\prime }\right|$, mitral valve center distance $\left|TM_{3\mathrm{DE}}-T^{\prime }M_{3\mathrm{DE}}^{\prime }\right|$, and outflow tract center $\left|TO_{3\mathrm{DE}}-T^{\prime }O_{3\mathrm{DE}}^{\prime }\right|$. The long-axis angle was calculated as the angle between the long axes in the original and repeated segmentations, i.e., the angle between the lines from $TM_{3\mathrm{DE}}$ and $T^{\prime }M_{3\mathrm{DE}}^{\prime }$ to $TA_{3\mathrm{DE}}$ and $T^{\prime }A_{3\mathrm{DE}}^{\prime }$. The transverse-plane angle was calculated as the angle between the outflow tracts in the original and repeated segmentations with respect to the long axis, i.e., the angle between $TO_{3\mathrm{DE}}$ and $T^{\prime }O_{3\mathrm{DE}}^{\prime }$ with respect to the line from $M_{\mathrm{CCTA}}$ to $A_{\mathrm{CCTA}}$.

Interobserver variability for 3DE segmentation as well as inter- and intraobserver variability for CCTA segmentation was quantified analogously.

### 2.7 Image Fusion and Visualization

For one of the included patients, the registration was visualized by displaying the coronary artery tree, obtained from CCTA, with the instantaneous longitudinal strain of the myocardium, obtained from 3DE.

3-D speckle tracking, including 3-D block matching method, was utilized to track and quantify myocardial wall motion in the 3DE volume. Instantaneous longitudinal strain was estimated using EchoPac 4D AutoLVQ (GE Healthcare, Horten, Norway). The estimated strain values at 337 points (14 longitudinal and 24 circumferential indices, as well as apex) in the myocardium at each time frame were exported. In the CCTA volume, the coronary artery tree was segmented by an experienced radiologist using Advantage Workstation.

## 3 Results

Table 1 summarizes the results of the chosen quality metrics for the three registration methods applied on datasets from 11 patients. Landmark distance magnitude is around 1 to 2 cm for all registration methods. Rotational measures are similar for landmark distance minimization and three-chamber alignment, but worse for ICP. ICP performs better with regard to mean point-to-point distance. The three-chamber Dice’s coefficient is similar among all methods.

**Table 1:** Quality measures for three registration methods (n=11).

	Landmark distance minimization	Iterative closest point	Three-chamber alignment
Apex distance (cm)	1.7±0.8	2.1±1.1	1.9±0.8
Mitral center distance (cm)	1.1±0.7	1.1±0.7	1.2±0.6
Aortic center distance (cm)	1.4±0.7	1.9±0.9	1.4±0.7
Long-axis angle (deg)	5.6±2.5	10.9±9.7	5.6±2.3
Transverse-plane angle (deg)	20.9±16.8	29.1±22.2	20.8±16.7
Mean point-to-point distance (cm)	0.47±0.15	0.41±0.10	0.48±0.15
Three-chamber Dice’s coefficient	0.85±0.08	0.84±0.10	0.85±0.08

**Fig. 4:**
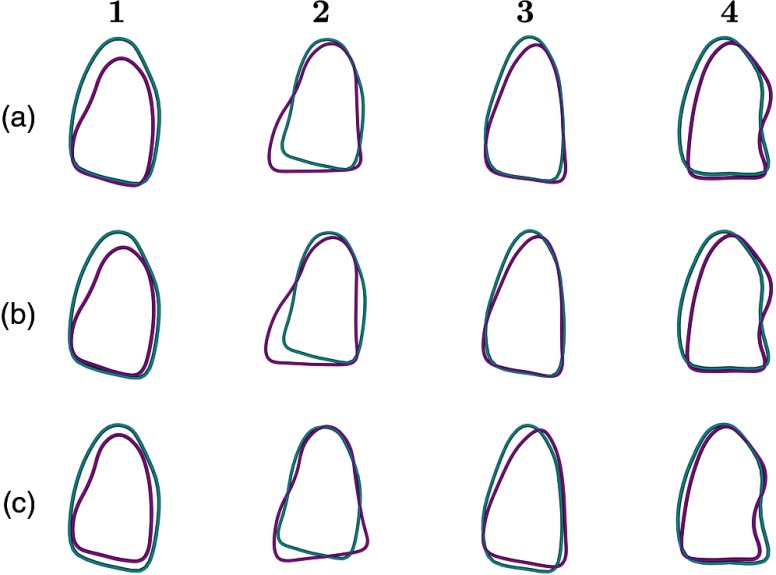
Three-chamber cross-sections of the segmented LV surfaces from CCTA (cyan) and 3DE (magenta), registered with (a) three-chamber alignment, (b) landmark distance minimization, and (c) LV surface ICP. Each column corresponds to one of four representative patients.

**Fig. 5:**
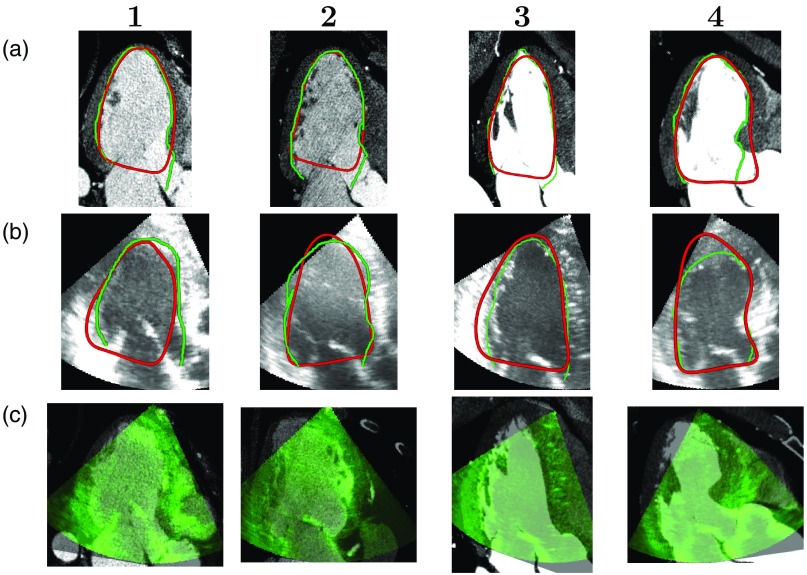
Row (a) shows three-chamber cross sections of CCTA images with semiautomatically segmented LV surfaces (red) and manually marked LV surfaces (green). Row (b) shows three-chamber cross sections of 3DE images with segmented (red) and manually marked (green) LV surfaces. Row (c) shows three-chamber cross sections of CCTA images with the same slice of the corresponding 3DE images overlaid. For row (c), images were registered using three-chamber alignment. Columns correspond to the columns of Fig. 4.

Figures 4(a)–4(c) give a qualitative view of the performance of the three evaluated registration methods for four representative patients by showing cross sections of the segmented endocardial surfaces from CCTA and 3DE, registered using the different methods. Figures 5(d) and 5(e) show cross sections of the semiautomatically segmented endocardial surfaces, used for registration, along with the manually marked endocardial surfaces, to give a qualitative view of the performance of the segmentation processes. Figure 5(f) overlays slices from CCTA with the corresponding slices from 3DE, as estimated using three-chamber alignment.

Statistical analysis on the quality measures presented in Table 1 was performed for each measure and each pair of registration methods for the entire population (n=11). Significant (p<0.05) difference was observed in six cases: between three-chamber alignment and landmark distance minimization with respect to apex distance (p=0.002), between landmark distance minimization and ICP with respect to aortic center distance (p=0.042), transverse-plane angle (p=0.014) and mean point-to-point distance (p=0.007), and between three-chamber alignment and ICP with respect to transverse-plane angle (p=0.010) and mean point-to-point distance (p=0.002).

Table 2 summarizes the inter- and intraobserver variability. The differences in landmark distance are on the order of 1 cm and smaller than the intermodality landmark distances as presented in Table 1. The inter- and intraobserver errors are similar.

**Table 2:** Intra- and interobserver variability. Segmentation was repeated for n=4 patients, once with the original operator and once with another operator. Repeated registration was performed with three-chamber alignment. Variability was quantified using differences in landmark positions and angles between original and repeated segmentations.

	Intraobserver	Interobserver
Repeated 3DE segmentations		
Apex distance (cm)	0.9±0.7	0.9±0.7
Aortic center distance (cm)	1.1±0.9	1.1±0.7
Mitral center distance (cm)	0.7±0.4	0.7±0.3
Long-axis angle (deg)	7.9±5.9	7.7±7.7
Transverse-plane angle (deg)	20.3±24.6	12.1±13.5
Repeated CCTA segmentations		
Apex distance (cm)	1.0±1.0	0.8±0.3
Aortic center distance (cm)	0.9±0.3	0.7±0.4
Mitral center distance (cm)	1.2±0.5	1.5±0.5
Long-axis angle (deg)	10.6±7.8	12.5±0.6
Transverse-plane angle (deg)	6.7±9.1	5.6±3.0

**Fig. 6:**
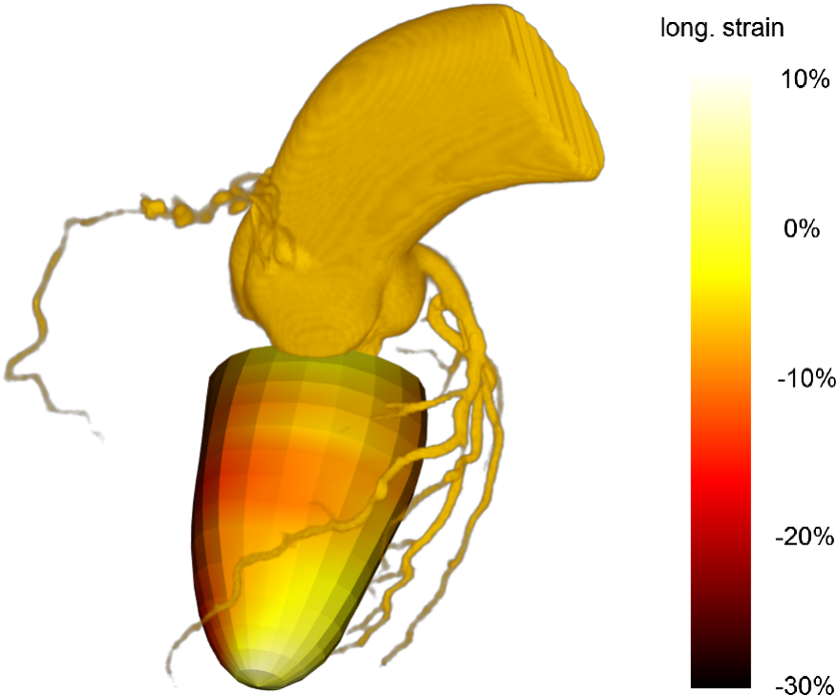
Fused image of the coronary artery tree from CCTA and the LV endocardium from 3DE in end-diastole. Instantaneous longitudinal strain is color-coded on the LV surface.

Figure 6 shows a fused image of anatomical information, the coronary artery tree from CCTA, and functional information, strain from 3DE, belonging to one of the included patients.

## 4 Discussion

This study aimed at comparing and validating three methods for automatic CCTA/3DE registration: landmark distance minimization, endocardium ICP alignment, and alignment of three-chamber views. All three registration methods showed good performance with regard to landmark distances, as seen in Table 1, with no obvious differences between the methods. Significant differences were found only between landmark distance minimization and three-chamber alignment with regard to apex distance (p=0.002) and between landmark distance minimization and ICP with regard to aortic center distance (p=0.042). Landmark distance minimization and three-chamber alignment performed better than ICP alignment with regard to the transverse-plane angle (p=0.014 and p=0.010, respectively). The anatomical landmarks used in landmark distance minimization and three-chamber alignment provide for more accurate rotational alignment, compared with ICP, which is likely the reason why the ICP method performed worse. The rotational component is crucial to properly relating LV segments and arteries. Furthermore, this result suggests that there was a difference in LV endocardial shape between CCTA and 3DE, caused by differences in actual shape or in segmented shape. Endocardium ICP alignment performed slightly better with regard to mean point-to-point distance, which is very similar to the metric minimized by the method (p=0.002 and p=0.007 when compared with three-chamber alignment and landmark distance minimization, respectively). This further indicates that a significant shape difference exists. Three-chamber Dice’s coefficient varied very little among methods.

Differences in actual shape are unavoidable due to the difference in the nature of CCTA and 3DE. In CCTA, the patient was medicated to decrease the heart rate, which likely reduced LV volume throughout the heart cycle,^24^ whereas no medication was given during 3DE examination. The 3DE and CCTA volumes were registered at the same temporal phase as measured in fraction of the R-R interval. However, changes in heart rate mostly affect the length of the diastolic phase,^25^ which may have resulted in a fusion of data from slightly different cardiac phases. The patient was also positioned differently during the two imaging examinations, which may be another reason for differences in LV volume and shape. 3DE was performed in the left lateral decubitus position, whereas the patient was positioned in the supine position during CCTA. Differences in respiratory motion may also have contributed to differences in LV shape and size. Due in part to these anatomical differences, perfect alignment of the two volumes would be impossible. Using an affine ICP algorithm^26^ would possibly result in better alignment of the LV shapes and thereby reduce registration errors. However, better alignment of LV shapes would not necessarily result in an improved correlation of stenotic arteries with functional abnormalities. The weak performance of the ICP method is more likely due to differences in LV orientation, which would not be improved using an affine ICP algorithm.

Cardiac image fusion systems combining CCTA with PET or SPECT have been shown to contribute to facilitated decision on revascularization strategy compared with side-by-side analysis^16^ and reduction of the frequency of subsequent revascularization procedures.^27^ Fusion of CCTA and 3DE has recently been shown to be feasible,^17^^,^^18^ but, to ascertain whether or not CCTA/3DE image fusion could be clinically viable and provide incremental value in CAD treatment decision, clinical trials must be performed. It is plausible that the registration accuracy shown here is adequate to correlate stenotic arteries with functional abnormalities in a clinical setting. The American Heart Association recommends dividing the LV into 17 segments when assessing myocardial function.^28^ In this model, the LV is divided into three levels along the long axis of the heart (apex, mid, and base). The basal and mid parts are then divided into six segments of 60 deg each, whereas the apical part is divided in four segments of 90 deg each and the apical cap, resulting in segments of an approximate size of 2  cm×2  cm. The segment size is thus larger than the registration errors of the proposed registration methods, which implies that the registration accuracy would likely be deemed acceptable for correlation of stenotic arteries with functional abnormalities in a clinical setting. Further, fusion of CCTA and SPECT has shown a registration accuracy of less than 10 mm^29^ and the clinical value of a similar fusion system has been demonstrated by increased specificity and positive predictive value compared with CCTA alone.^30^ Registration accuracy for CCTA/SPECT fusion is in the same magnitude but slightly smaller than the demonstrated registration accuracy in this study, which probably is related to larger differences in LV shape between CCTA and 3DE than between SPECT and CT. Still, these results indicate that the methods proposed in this study may also have potential for clinical use.

Patients with complex CAD and multivessel disease are frequently referred to revascularization procedures, although studies have shown that stenoses found on their angiograms often are functionally insignificant.^31^ Cardiac image fusion may be beneficial for this patient group by facilitating identification of the culprit lesion responsible for perfusion defect and avoiding invasive procedures with risk of adverse effects.

A benefit of using 3DE as functional modality in cardiac image fusion instead of SPECT or PET is the lack of additional ionizing radiation. Still, the individual radiation exposure needs to be minimized and the risk versus benefit of each imaging examination must be taken into account. Over the last years, CCTA has emerged as a promising noninvasive alternative to invasive coronary angiography due to improved spatial resolution and lower radiation dose. The conventional way of analyzing coronary artery anatomy is by curved multiplanar 2-D slices in a 3-D CCTA volume. 3-D techniques alone have demonstrated insufficient performance in the evaluation of stenosis^32^ and need further development or to be used in combination with 2-D techniques. In cardiac image fusion, 3-D visualization of the coronary artery tree has the advantage of allowing for easier spatial assignment of stenosis and dysfunctional myocardium. A possible future extension of the proposed fusion method is to include simulated FFR measurements from CCTA as a measure of hemodynamic relevance of a stenosis.^33^

A limitation of this study is the small group of patients enrolled and only one female patient was included. This was partly due to lack of female patients with suspected CAD undergoing CCTA on clinical indication in our clinic. However, this study aimed at comparing and demonstrating the potential of different registration methods for automatic CCTA/3DE registration. For this purpose, the included patient group was considered to properly represent a variety of cardiac anatomies present in a patient population with known or suspected CAD. The clinical effectiveness of the proposed fusion technique still needs to be determined in larger studies of both female and male patient groups.

The registration methods presented depended on proper segmentation since the registration is based on alignment of either landmarks or surfaces determined by the segmentation. These segmentation algorithms have been validated in previous studies with credible results.^34^^,^^35^ However, the registration methods are semiautomatic and hence user-dependent since manual initialization is required for the segmentation step. Therefore, the intra- and interobserver variability for repeated segmentations was investigated and found to be overall smaller than the corresponding registration errors. This variation was considered to be acceptable and unlikely to be significant in a clinical setting. Fully automatic segmentation algorithms,^36^^,^^37^ which could be used to further eliminate the user dependency in the registration process, also exist. However, the proposed semiautomatic approach is more time-efficient and likely more robust compared with a manual registration process, with a total processing time of ∼20  min per patient. Optimization of user interfaces could further reduce processing time to about 5 min per patient.

Myocardial strain assessment has been shown to be useful in the detection of hemodynamically significant stenosis, in particular, when performing stress echocardiography.^38^ The proposed fusion could be extended to include functional parameters from stress echocardiography by adding registration of baseline and stress echocardiography. In addition to myocardial strain assessment, stress echocardiography enables real-time quantification of myocardial perfusion, which has shown promising results in the evaluation of myocardial ischemia in different patient populations and settings.^39^^,^^40^ However, the number of clinical studies presented is still relatively small and mostly limited to two-dimensional views of the myocardium. Further development is thus needed before presenting reliable volumetric perfusion data as a functional parameter in the proposed cardiac image fusion. The optimal parameter or combination of functional parameters from 3DE to be visualized in the fusion needs to be investigated in further studies.

Traditionally, fusion of morphological and functional information is performed by mental integration that relies on models allocating myocardial segments to specific coronary arteries.^41^ However, these models have been shown to poorly assign myocardial segments with coronary arteries because of a highly varying coronary anatomy among individuals.^42^ The 3-D visualization approach proposed in this study allows for improved assignment of arteries and corresponding myocardial segments. This could be further facilitated by automatic visualization of a coronary artery and its most probable perfused myocardial segment. The feasibility of such visualization has not been investigated in the present study.

The registration of CCTA/3DE data was performed in a single time frame, 35% or 75% of the R-R interval. The CCTA was performed in these time frames since the heart is moving at least at end systole (35% of R-R interval) and middiastole (75% of R-R interval) in patients with heart rates below 80 beats per minute.^43^ A limitation of using CCTA data from a single time frame is that the coronary artery tree remains static while the functional parameter from 3DE is visualized with high temporal resolution. This can lead to additional spatial misalignment, since the coronary artery motion has been shown to vary with heart rate and throughout the cardiac cycle.^43^ The use of multiphase CCTA in future versions of the fusion technique would thus most likely improve the alignment of coronary arteries and myocardial segments throughout the entire cardiac cycle.

## 5 Conclusion

Three methods for registration of CCTA and 3DE volumes were implemented and compared on data from patients with suspected CAD. All methods provided adequate registration and performed well with regard to landmark distances. Landmark distance minimization and alignment of the three-chamber planes performed better with regard to LV rotation than minimization of distances between endocardial surfaces (ICP). However, clinical studies must be performed to ascertain whether or not CCTA/3DE image fusion could be clinically viable and provide incremental value in CAD treatment decision.

## References

[1] Finegold J. A. Asaria P. Francis D. P., “Mortality from ischaemic heart disease by country, region, and age: statistics from World Health Organisation and United Nations,” Int. J. Cardiol. 168(2), 934–945 (2013). IJCDD5 0167-5273 10.1016/j.ijcard.2012.10.046 23218570 PMC3819990

[2] Murray C. J. Lopez A. D., “Mortality by cause for eight regions of the world: global burden of disease study,” Lancet 349(9061), 1269–1276 (1997). LANCAO 0140-6736 10.1016/S0140-6736(96)07493-4 9142060

[3] Montalescot G. et al., “2013 ESC guidelines on the management of stable coronary artery disease: the task force on the management of stable coronary artery disease of the European society of cardiology,” Eur. Heart J. 34(38), 2949–3003 (2013). EHJODF 0195-668X 10.1093/eurheartj/eht296 23996286

[4] Keeley E. C. Boura J. A. Grines C. L., “Comparison of primary and facilitated percutaneous coronary interventions for ST-elevation myocardial infarction: quantitative review of randomised trials,” Lancet 367(9510), 579–588 (2006). LANCAO 0140-6736 10.1016/S0140-6736(06)68148-8 16488801

[5] Pijls N. H. et al., “Fractional flow reserve versus angiography for guiding percutaneous coronary intervention in patients with multivessel coronary artery disease: 2-year follow-up of the FAME (fractional flow reserve versus angiography for multivessel evaluation) study,” J. Am. Coll. Cardiol. 56(3), 177–184 (2010). JACCDI 0735-1097 10.1016/j.jacc.2010.04.012 20537493

[6] Windecker S. et al., “2014 ESC/EACTS guidelines on myocardial revascularization: the task force on myocardial revascularization of the European Society of Cardiology (ESC) and the European Association for Cardio-Thoracic Surgery (EACTS),” Eur. Heart J. 35(37), 2541–2619 (2014). EHJODF 0195-668X 10.1093/eurheartj/ehu278 25173339

[r7] Schroeder S. et al., “Cardiac computed tomography: indications, applications, limitations, and training requirements report of a writing group deployed by the working group nuclear cardiology and cardiac CT of the European Society of Cardiology and the European Council of Nuclear Cardiology,” Eur. Heart J. 29(4), 531–556 (2008). EHJODF 0195-668X 10.1093/eurheartj/ehm544 18084017

[8] Schuijf J. D. et al., “Cardiac imaging in coronary artery disease: differing modalities,” Heart 91(8), 1110–1117 (2005). 10.1136/hrt.2005.061408 16020614 PMC1769025

[9] Lindstaedt M. et al., “How good are experienced interventional cardiologists at predicting the functional significance of intermediate or equivocal left main coronary artery stenoses?” Int. J. Cardiol. 120(2), 254–261 (2007). IJCDD5 0167-5273 10.1016/j.ijcard.2006.11.220 17346818

[10] Underwood S. et al., “Myocardial perfusion scintigraphy: the evidence,” Eur. J. Nucl. Med. Mol. Imaging 31(2), 261–291 (2004). 10.1007/s00259-003-1344-5 15129710 PMC2562441

[11] Nandalur K. R. et al., “Diagnostic performance of positron emission tomography in the detection of coronary artery disease: a meta-analysis,” Acad. Radiol. 15(4), 444–451 (2008). 10.1016/j.acra.2007.08.012 18342769

[12] Paetsch I. et al., “Comparison of dobutamine stress magnetic resonance, adenosine stress magnetic resonance, and adenosine stress magnetic resonance perfusion,” Circulation 110(7), 835–842 (2004). CIRCAZ 0009-7322 10.1161/01.CIR.0000138927.00357.FB 15289384

[13] Togni M. et al., “Percutaneous coronary interventions in Europe 1992–2001,” Eur. Heart J. 25(14), 1208–1213 (2004). EHJODF 0195-668X 10.1016/j.ehj.2004.04.024 15246638

[14] Schindler T. H. et al., “Fusion imaging: combined visualization of 3D reconstructed coronary artery tree and 3D myocardial scintigraphic image in coronary artery disease,” Int. J. Card. Imaging 15(5), 357–368 (1999). 10.1023/A:1006232407637 10595402

[15] Kalbfleisch H. Hort W., “Quantitative study on the size of coronary artery supplying areas postmortem,” Am. Heart J. 94(2), 183–188 (1977). 10.1016/S0002-8703(77)80278-0 141876

[16] Gaemperli O. et al., “Cardiac image fusion from stand-alone SPECT and CT: clinical experience,” J. Nucl. Med. 48(5), 696–703 (2007). JNMEAQ 0161-5505 10.2967/jnumed.106.037606 17475956

[17] Lipiec P. et al., “Fusion of morphological data obtained by coronary computed tomography angiography with quantitative echocardiographic data on regional myocardial function,” Cardiol. J. 23(3), 264–269 (2016). 10.5603/CJ.a2016.0015 27064799

[18] Maffessanti F. et al., “Fusion of coronary anatomy from computed tomography with left ventricular strain function from 3D echocardiography,” Circulation 130(Suppl. 2), A19406–A19406 (2014). CIRCAZ 0009-7322

[19] Nordenfur T. et al., “Algorithm comparison for cardiac image fusion of coronary computed tomography angiography and 3D echocardiography,” in IEEE Int. Ultrasonics Symp. (IUS ’15), pp. 1–4, IEEE (2015).

[20] Orderud F., “Real-time segmentation of 3D echocardiograms using a state estimation approach with deformable models,” Doctoral thesis, Norwegian University of Science and Technology, Trondheim (2010).

[21] Gower J. C., “Generalized procrustes analysis,” Psychometrika 40(1), 33–51 (1975).0033-3123 10.1007/BF02291478

[22] Zhang Z., “Iterative point matching for registration of free-form curves and surfaces,” Int. J. Comput. Vision 13(2), 119–152 (1994). IJCVEQ 0920-5691 10.1007/BF01427149

[23] Schroeder W. Martin K. Lorensen B., The Visualization Toolkit, 4th ed., Kitware, Inc., Clifton Park, New York (2006).

[24] Lee S. J. Sung Y. K. Zaragoza A. J., “Effects of nitroglycerin on left ventricular volumes and wall tension in patients with ischaemic heart disease,” Heart 32(6), 790–794 (1970). 10.1136/hrt.32.6.790 PMC487416 5006415

[25] Husmann L. et al., “Coronary artery motion and cardiac phases: dependency on heart rate– implications for CT image reconstruction,” Radiology 245(2), 567–576 (2007). RADLAX 0033-8419 10.1148/radiol.2451061791 17848683

[26] Du S. et al., “Affine iterative closest point algorithm for point set registration,” Pattern Recognit. Lett. 31(9), 791–799 (2010). PRLEDG 0167-8655 10.1016/j.patrec.2010.01.020

[27] Pazhenkottil A. P. et al., “Impact of cardiac hybrid single-photon emission computed tomography/computed tomography imaging on choice of treatment strategy in coronary artery disease,” Eur. Heart J. 32(22), 2824–2829 (2011). 10.1093/eurheartj/ehr232 21804107 PMC3214723

[28] Cerqueira M. D. et al., “Standardized myocardial segmentation and nomenclature for tomographic imaging of the heart,” Circulation 105(4), 539–542 (2002). CIRCAZ 0009-7322 10.1161/hc0402.102975 11815441

[29] Woo J. et al., “Geometric feature-based multimodal image registration of contrast-enhanced cardiac CT with gated myocardial perfusion SPECT,” Med. Phys. 36(12), 5467–5479 (2009). MPHYA6 0094-2405 10.1118/1.3253301 20095259 PMC4108676

[30] Rispler S. et al., “Integrated single-photon emission computed tomography and computed tomography coronary angiography for the assessment of hemodynamically significant coronary artery lesions,” J. Am. Coll. Cardiol. 49(10), 1059–1067 (2007). JACCDI 0735-1097 10.1016/j.jacc.2006.10.069 17349885

[31] Tonino P. A. et al., “Angiographic versus functional severity of coronary artery stenoses in the fame study fractional flow reserve versus angiography in multivessel evaluation,” J. Am. Coll. Cardiol. 55(25), 2816–2821 (2010). 10.1016/j.jacc.2009.11.096 20579537

[32] Wang C. et al., “Can segmented 3D images be used for stenosis evaluation in coronary CT angiography?” Acta Radiol. 53(8), 845–851 (2012). 10.1258/ar.2012.120053 22855418

[33] Kim H. J. et al., “Patient-specific modeling of blood flow and pressure in human coronary arteries,” Ann. Biomed. Eng. 38(10), 3195–3209 (2010). 10.1007/s10439-010-0083-6 20559732

[34] Orderud F. et al., “Combining edge detection with speckle-tracking for cardiac strain assessment in 3D echocardiography,” in Ultrasonics Symp. (IUS ’08), IEEE (2008). 10.1109/ULTSYM.2008.0483

[35] Orderud F. et al., “Real-time left ventricular speckle-tracking in 3D echocardiography with deformable subdivision surfaces,” in MICCAI 2008 Workshop on Analysis of Functional Medical Images (2008).

[36] Barbosa D. et al., “Fast and fully automatic 3-D echocardiographic segmentation using B-spline explicit active surfaces: feasibility study and validation in a clinical setting,” Ultrasound Med. Biol. 39(1), 89–101 (2013). 10.1016/j.ultrasmedbio.2012.08.008 23200179

[37] Santiago C. Nascimento J. C. Marques J. S., “Automatic 3-D segmentation of endocardial border of the left ventricle from ultrasound images,” IEEE J. Biomed. Health Inform. 19(1), 339–348 (2015). 10.1109/JBHI.2014.2308424 25561455

[38] Uusitalo V. et al., “Two-dimensional speckle-tracking during dobutamine stress echocardiography in the detection of myocardial ischemia in patients with suspected coronary artery disease,” J. Am. Soc. Echocardiography 29(5), 470–479 (2016). 10.1016/j.echo.2015.12.013 26852941

[39] Mor-Avi V. et al., “Combined assessment of myocardial perfusion and regional left ventricular function by analysis of contrast-enhanced power modulation images,” Circulation 104(3), 352–357 (2001). CIRCAZ 0009-7322 10.1161/01.CIR.104.3.352 11457757

[40] Winter R. Gudmundsson P. Willenheimer R., “Real-time perfusion adenosine stress echocardiography in the coronary care unit: a feasible bedside tool for predicting coronary artery stenosis in patients with acute coronary syndrome,” Eur. J. Echocardiography 6(1), 31–40 (2005). 10.1016/j.euje.2004.06.003 15664551

[41] Cerqueira M. D. et al., “Standardized myocardial segmentation and nomenclature for tomographic imaging of the heart. A statement for healthcare professionals from the cardiac imaging committee of the council on clinical cardiology of the american heart association,” Int. J. Cardiovasc. Imaging 18(1), 539–542 (2002). 10.1161/hc0402.102975 12135124

[42] Javadi M. S. et al., “Definition of vascular territories on myocardial perfusion images by integration with true coronary anatomy: a hybrid pet/ct analysis,” J. Nucl. Med. 51(2), 198–203 (2010). 10.2967/jnumed.109.067488 20080895

[43] Lu B. et al., “Coronary artery motion during the cardiac cycle and optimal ECG triggering for coronary artery imaging,” Invest. Radiol. 36(5), 250–256 (2001). 10.1097/00004424-200105000-00002 11323512

